# Daily Activity of the Housefly, *Musca domestica*, Is Influenced by Temperature Independent of 3′ UTR *period* Gene Splicing

**DOI:** 10.1534/g3.117.042374

**Published:** 2017-06-15

**Authors:** Olga Bazalova, David Dolezel

**Affiliations:** * Biology Center, Czech Academy of Sciences, 37005 České Budějovice, Czech Republic; † Department of Molecular Biology, Faculty of Sciences, University of South Bohemia, 37005 České Budějovice, Czech Republic

**Keywords:** temperature compensation of circadian rhythms, locomotor activity, transcription, mRNA splicing, circadian clock genes

## Abstract

Circadian clocks orchestrate daily activity patterns and free running periods of locomotor activity under constant conditions. While the first often depends on temperature, the latter is temperature-compensated over a physiologically relevant range. Here, we explored the locomotor activity of the temperate housefly *Musca domestica*. Under low temperatures, activity was centered round a major and broad afternoon peak, while high temperatures resulted in activity throughout the photophase with a mild midday depression, which was especially pronounced in males exposed to long photoperiods. While *period* (*per*) mRNA peaked earlier under low temperatures, no temperature-dependent splicing of the last *per* 3ʹ end intron was identified. The expression of *timeless*, *vrille*, and *Par domain protein 1* was also influenced by temperature, each in a different manner. Our data indicated that comparable behavioral trends in daily activity distribution have evolved in *Drosophila melanogaster* and *M. domestica*, yet the behaviors of these two species are orchestrated by different molecular mechanisms.

Circadian clocks are ubiquitous adaptations to periodic day/night alternations and orchestrate metabolic and behavioral activities of many organisms. For instance, *Drosophila melanogaster* displays a bimodal locomotor activity pattern with morning and evening peaks separated by a siesta under a constant temperature (Rosato and Kyriacou 2006) or afternoon activity bursts under natural environmental conditions (Vanin *et al.* 2012; Green *et al.* 2015). Under elevated temperatures, the morning peak is advanced and the evening peak is delayed. In contrast, the overall free running period under constant conditions is temperature-compensated over a physiologically relevant range. Circadian genes drive both of these activities.

Temperature strongly affects the weak splice site of the last *period* (*per*) gene intron (dmpi8) and locomotor activity pattern of *D. melanogaster* (Majercak *et al.* 1999; Collins *et al.* 2004; Majercak *et al.* 2004). However, a comparison of two tropical *Drosophila* species, *D. yakuba* and *D. santomea*, revealed that temperature has no effect on the splicing efficiency of the last *per* intron, nor on the daily activity of the flies (Low *et al.* 2008). Transgenic constructs specifically addressed the structure of this intron and established its splicing efficiency as an important molecular mechanism adjusting behavior to the temperature. The impact of temperature on the transcription of *per* and *timeless* (*tim*) was explored both in *D. melanogaster* exposed to a laboratory-controlled environment (Boothroyd *et al.* 2007) and more recently, under natural conditions (Montelli *et al.* 2015).

Notably, the locomotor activity of northern *Drosophila* species, including *D. littoralis* and *D. montana*, is tightly linked to weather, and their behavior becomes arrhythmic under constant dark conditions (Lankinen 1986; Kauranen *et al.* 2012; D. Dolezel and J.C. Hall, unpublished data). Given these interspecific differences within the *Drosophila* genus, we decided to explore the impact of temperature on the locomotor activity of the housefly, *Musca domestica*, a dipteran species using a characterized circadian toolkit (Codd *et al.* 2007), including rescue experiments of the *D. melanogaster per*^0^ mutant (Piccin *et al.* 2000) and numerous anatomical data (Pyza and Meinertzhagen 1997; Miskiewicz *et al.* 2008).

## Materials and Methods

### Fly maintenance

*M. domestica* larvae were raised on wheat bran (55 g), heat-inactivated yeast (3 g), milk (150 ml), and antimycotic nipagin (0.35 g; Sigma) until pupariation. After eclosion, adult flies were fed water, sugar, and dried milk. Fly stocks were maintained at 25° under either 12 hr light: 12 hr dark cycles (LD 12:12) or 16 hr light: 8 hr dark cycles (LD 16:8). In our study, we mainly used homozygous strains of *white eye* and *apterous* mutations (gift from Daniel Bopp, University of Zurich) (Hediger *et al.* 2001; Codd *et al.* 2007). For behavioral experiments, we also collected a wild-type strain in České Budějovice (Czech Republic), which was amplified for three to five generations, and used it for locomotor activity recordings. The species identity was verified by sequencing the intron of the *per* gene.

### Locomotor activity

Male and female flies, 3–5 d old, were individually housed in glass test tubes (24 × 150 mm, PYREX; Sigma) with a sugar lump wrapped in a mesh bag on one side and water reservoir on the other side. The movements of the flies were automatically detected using an infrared photosensor (Large Activity Monitors, LAM; TriKinetics, Waltham, MA) and recorded at 5 min intervals. Monitors were placed under a controlled regime in MIR 153 or 253 incubators (SANYO/Panasonic) with a 15 W cool white lamp (ZS15; Top Light) generating light at ∼400–800 lux.

To determine the free running period, flies were entrained for 4 d at either LD 16:8, LD 12:12, or LD 8:16 (at 15, 25, or 35°) and released into a constant, dark (DD) environment of the same temperature for another 10 d. The activity data were evaluated using ActogramJ software (Schmid *et al.* 2011).

Daily patterns of locomotor activity were recorded at three different, constant temperatures (15, 25, or 35°) under either LD 16:8 or LD 12:12 regimes. Data were collected for at least 14 d, but the last 4 d served as a control of survivor and were omitted from the analysis. To compare daily activity distribution patterns, data were collected in 5 min bins, smoothed by factor 2 in Actogram J (Schmid *et al.* 2011), and an activity histogram was plotted as mean ± SEM in Graphpad Prism. To compare absolute activity levels under different conditions for each individual fly, the total average activity at 24 hr intervals was calculated from day 5 to 14 and presented as one point (Graphpad Prism).

### Gene cloning

To collect samples for cDNA cloning, the flies were snap-frozen and their heads were either stored at −80°, or immediately used for total RNA isolation with the TRIzol-reagent (Ambion). Using the oligo (dT) primer (24mer) and Superscript III reverse transcriptase (Invitrogen), 5 μg of total RNA was reverse transcribed. Sequences for *M. domestica* circadian clock genes, *casein kinase 2beta* (*Md_ck 2beta*), *clockwork orange* (*Md_cwo*), and *Par domain protein 1* (*Md_Pdp1*), were obtained using PCR with degenerate primers designed according to the conserved protein regions of insect (*D. melanogaster*, *Apis melifera*, *Culex pipiens*, *Aedes aegypti*, *Danaus plexipus*, *Tribolium castaneum*), amphibian (*Xenopus laevis*), and mammalian (*Rattus norvegicus*, *Homo sapiens sapiens*) clock gene orthologs. Short fragments (200–700 bp) were amplified, cloned, and sequenced. Gene sequences were further extended by primer walking 3ʹ - RACE (rapid amplification of 3ʹ ends) and 5ʹ - RACE (Ambion). The sequences of the *Musca pigment dispersing factors* (*Md_pdf*) (Matsushima *et al.* 2004), *period* (Piccin *et al.* 2000), *vrille* (*Md_vri*), *Clock* (*Md_Clk*), *cycle* (*Md_cyc*), and *timeless* (*Md_tim*) (Codd *et al.* 2007) have been previously described. The intron sequences close to the coding sequence (CDS) 3ʹ end of the *cwo*, *ck 2beta*, and *pdf* genes and close to the start codon of the *Pdp 1_epsilon_* gene were obtained from genomic DNA using specific primers. No intron sequences were identified in the *double time* gene. All primers used are listed in Supplemental Material, Table S1.

### Phylogenetic analysis

To clarify which *cryptochromes* are encoded in the *Musca* genome, CRY proteins were aligned and trimmed using the MAFFT program (Geneious; Biomatters) and substitution models were tested in the ProTest 2.4 program (Abascal *et al.* 2005). Phylogenetic trees were constructed under the WAG and LG models (RaXML) and in the Fasttree algorithm (Geneious 8.1; Biomatters). Since all trees shared the same topology, we only presented phylogenetic analyses performed in RaXML 7.2.8 under the LG+Gamma model with 500 bootstrap replications.

As a reference, we used insects containing both mammalian- and *Drosophila*-type CRYs: *Anopheles gambiae*, *D. plexippus* (Yuan *et al.* 2007), *Blattella germanica* (Bazalova *et al.* 2016), *Pogonus chalceus*, insect with only mammalian CRYs: *A. mellifera* (Rubin *et al.* 2006) and *Pyrrhocoris apterus* (Bajgar *et al.* 2013b), and *D. melanogaster* representing insects with only the photosensitive CRY (Stanewsky *et al.* 1998).

### Expression analysis

Three independent animal groups were reared and killed for each experiment. For each experiment, 5- to 10-d-old males and females were collected separately, and their heads were stored at −80° until needed for RNA extraction. Total RNA was isolated from 25 heads per time-point using the TRIzol-reagent (Ambion) according to the manufacturer’s instructions. Using oligo (dT) primers and Superscript III reverse transcriptase (Invitrogen), 1 μg of total RNA was reverse transcribed. To safeguard against the amplification of possible contaminant genomic DNA, the primers were designed either to anneal only to a template corresponding to the spliced transcript, or (in the case of *dbt*) the genomic DNA was removed from the sample using TURBO DNase (TURBO DNA-free Kit; Ambion), according to the manufacturer’s instructions, or (in the case of *tim*) the primers were designed to include a large (2.5 kb) intron in the genomic DNA template (Codd *et al.* 2007).

In total, 3 μl of 100× times (*M. domestica*) or 3 μl of 50× times (*D. melanogaster*) diluted complementary DNA (cDNA) was used for the 12 μl PCR reaction (either in IQ SYBRGreen Supermix from Bio-Rad; or with 2×TP SYBR Master Mix from Top-Bio; primers 0.4 μM each). Amplifications were carried out on the C1000 Thermal Cycler (Bio-Rad) in 96-well micro plates (Hard-Shell; Bio-Rad) according to the following protocol: initial denaturation (94°, 2 min) followed by 35 cycles of denaturation (94°, 10 sec), annealing primers (60°, 20 sec), and elongation (72°, 20 sec). Product size was always confirmed by the melting analysis. Each cDNA sample was amplified in triplicates for each primer combination, where *rp49* served as a normalization reference. A list of the specifications of the primers used is given in Table S1. Data were analyzed and quantified using CFX Manager Software. Relative values were standardized to *rp49* and normalized to the sample with the highest expression (Kobelkova *et al.* 2010). Values represent the mean of three independent biological replicates ± SD.

### Promoter comparison

Promoters of *D. melanogaster* and *M. domestica* were explored manually to identify conserved *cis*-regulatory motifs that were subsequently highlighted as sequence annotations in Geneious 7.1 (Biomatters). Corresponding figures were manually redrawn in Adobe Illustrator CS5. The expected frequencies of particular *cis*-motifs were compared to observed frequencies in a Microsoft Excel 2010 spreadsheet, obtained values were color-coded (Conditional formatting in Excel), and the figure was finalized in Adobe Illustrator.

### Data availability

*Drosophila* strain is available on request from our laboratory. *Musca* strain was obtained from D. Bopp (Hediger *et al.* 2001). Sequence data of *D. melanogaster* and *M. domestica* circadian genes can be found on FlyBase (http://flybase.org/) and VectorBase (https://www.vectorbase.org/) databases, respectively (see Table S2 for accession numbers).

## Results

### Temperature compensation of the free running period

**Figure 1:**
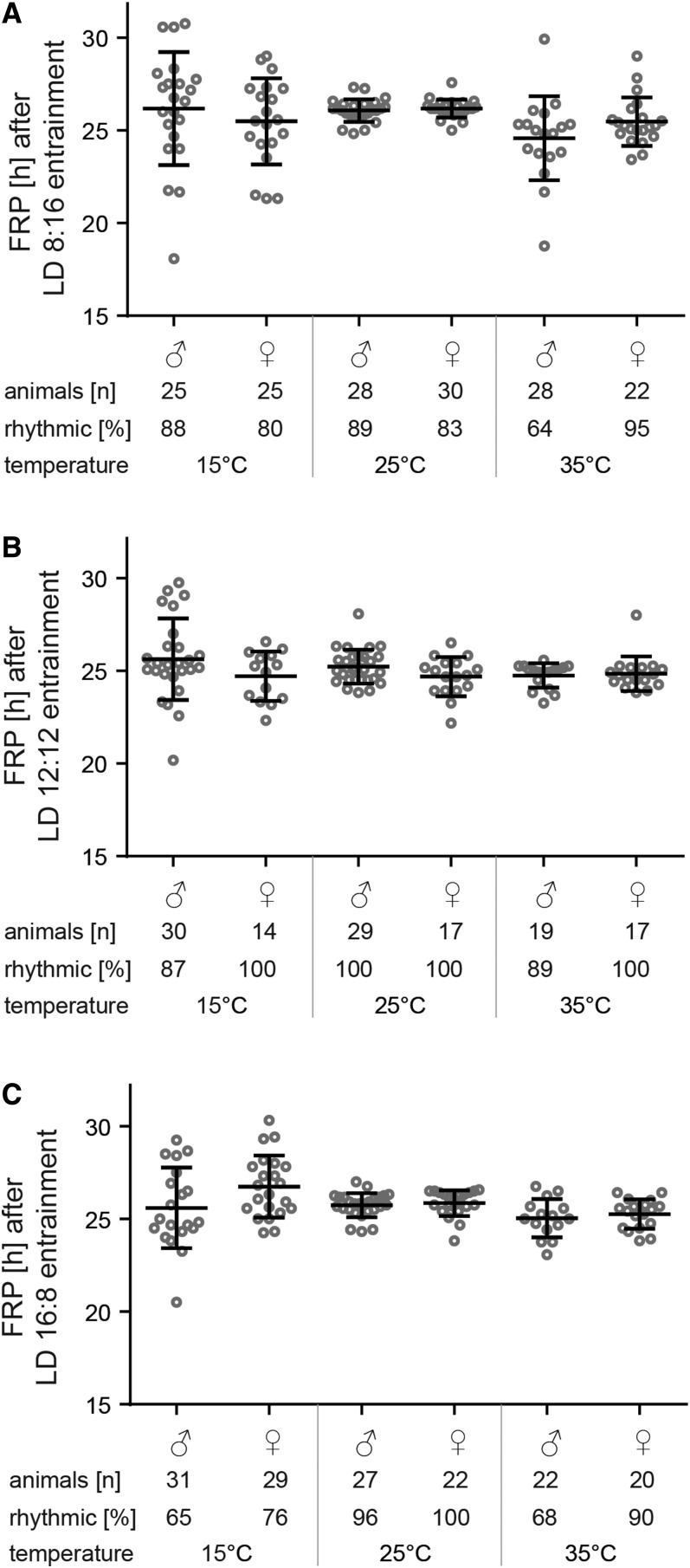
Free running period of males and females (±SD) at 15, 25, and 35° after entrainment at three different photoperiods: (A) short day LD 8:16, (B) equinox LD 12:12, and (C) long day LD 16:8. Each dot corresponds to individual flies. Number of all animals measured and percent of rhythmic individuals is shown under each chart.

Our first goal was to verify if the circadian clock of *M. domestica* was functional at all temperatures. Flies were entrained to three different photoperiods, at three different temperatures (nine different combinations), and afterward released to DD. In general, behavioral rhythmicity is well temperature-compensated and free running period stayed constant at all three different temperatures (Figure 1). While the free running period was observed in nearly all flies exposed to 25°, both lower (15°) and higher (35°) temperatures resulted in a lower percentage of rhythmic individuals, particularly males, and a higher variability in the free running period of rhythmic individuals (Figure 1). Moreover, a short photoperiod entrainment (LD 8:16) slightly reduced the percentage of rhythmic individuals even at 25° (Figure 1A). Examples of double-plotted actograms are shown in Figure S1.

### Locomotor activity distribution in LD

**Figure 2:**
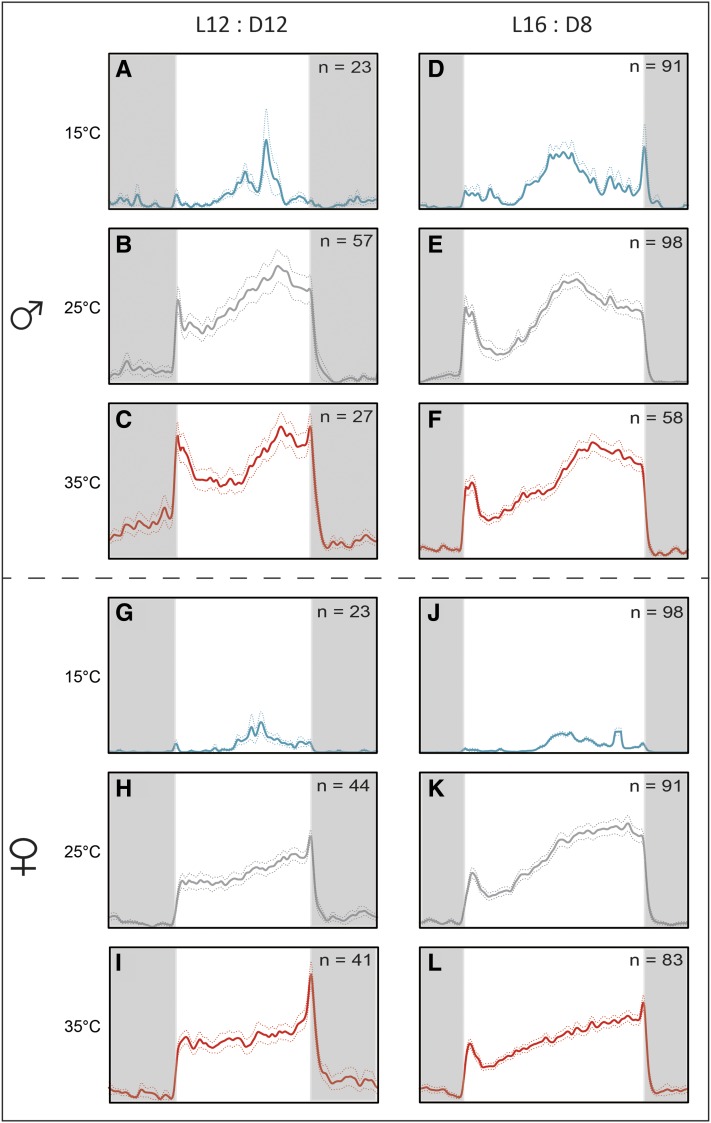
Locomotor activity pattern of *M. domestica* kept in LD 12:12 (left column) and LD 16:8 (right column). Blue, gray, and red lines represent 15° (low, panels A, D, G, J), 25° (normal, panels B, E, H, K), and 35° (high, panels C, F, I, L) temperatures, respectively. Flies were measured individually. The average ± SD is shown as dashed colored lines; *n*, numbers of animals used in the experiment. Vertical dashed gray line corresponds to ZT 12 under the LD 16:8 regime.

**Figure 3:**
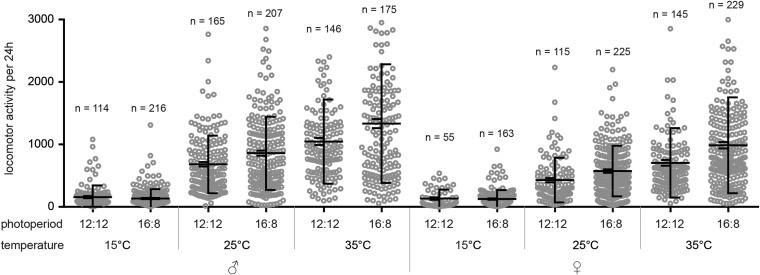
Locomotor activity levels of *M. domestica* plotted as the number of laser beam crosses per 24 hr. The left side depicts the mean ± SEM; the right side depicts the average ± SD. Each circle corresponds to individual flies, and number of analyzed days is shown above each category. See Table S3 for statistics.

We explored the impacts of temperature and photoperiod on daily activity patterns. In general, a low temperature of 15° resulted in locomotor activity being centered around the middle of the photophase (Figure 2, A, D, G, and J) in both photoperiods, whereas at 25 and 35°, flies were active during the entire photophase (Figure 2, B, C, E, F, H, I, K, and L). There was also a mild difference between male and female activity at 25 and 35°. Males showed a slightly bimodal pattern with the light on peak followed by a mild trough in the first half of the photophase, then a gradual activity increase during the afternoon reaching a maximum ∼10 hr after the light on signal (Figure 2, B, C, E, and F). The afternoon activity peak was delayed with the temperature increase (compare Figure 2, A–C for the short day and Figure 2, D–F for the long day). The activity gradually decreased during the second half of the photophase under the long photoperiod of LD 16:8 (Figure 2, E and F). In contrast, the activity of females increased progressively during the photophase at 25 and 35° peaking either after the light off signal (Figure 2, H, I, and L) or just at the end of the photophase (Figure 2K). The overall activity at 15° was markedly lower than the activity detected at 25 or 35° in both sexes (Figure 3 and Table S3). At 25 and 35°, males were slightly more active than females (Figure 3 and Table S3).

### Cryptochrome gene in Musca: phylogenetic analysis

**Figure 4:**
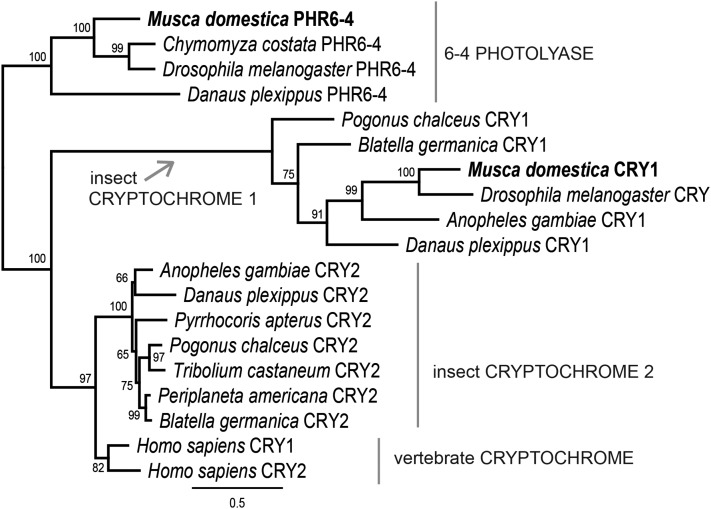
Phylogeny of *M. domestica* CRY in context of insect CRYs. The tree was obtained from the RAxML 7.2.8 analysis of protein sequence alignment under the LG+Gamma substitutional model. Bootstrap support from 500 replicates is shown in percent under each node. 6–4 photolyases served as an outgroup.

The initial search identified two *cry*-like genes in the *M. domestica* genome. Phylogenetic analysis revealed three clearly distinguished clusters: (i) 6–4 photolyase, (ii) insect cryptochrome 1 (*Drosophila*-type CRYs), and (iii) a group containing both human CRYs and insects CRY2 (Figure 4). This analysis, unambiguously supported by bootstrap values, completely agreed with the previously published phylogeny of CRYs (Yuan *et al.* 2007). Thus, *M. domestica* contained one CRY protein of *Drosophila*-type (Md*_*CRY1) and the second gene clearly belonged to 6–4 photolyases (Figure 4).

Our phylogenetic analysis revealed an additional, interesting feature. In an attempt to add CRY proteins from relevant holometabolan insects, we sought transcriptomes of beetles (Coleoptera). Whereas in *T. castaneum* only one gene belonging to the CRY2 group was identified, in another beetle, *P. chalceus*, both the CRY1 (*Drosophila*-type) and CRY2 -coding genes were found (Figure 4). This finding supports the remarkable diversity of the circadian clock design, particularly the role of various CRY combinations reported in holometabola (Yuan *et al.* 2007) and recently in hemimetabola (Bazalova *et al.* 2016).

Given the noncyclical expression of *Md_cry1* mRNA (Codd *et al.* 2007), which is in contrast with the expression of *Drosophila cry* (*Dm_cry*) (Emery *et al.* 1998), and the possible role of *Dm_cry* in temperature-dependent rhythmicity (Dolezelova *et al.* 2007), we sought to analyze the expression profile of both *Md_cry1* and *Md_phr6-4*.

### mRNA expression at different temperatures: M. domestica

qRT PCR was performed on samples isolated from male *M. domestica* heads collected every 2 hr. First, we explored expression of circadian clock genes under the LD and DD conditions at 25° (Figure S2). Cyclically expressed genes were further analyzed at three different temperatures.

**Figure 5:**
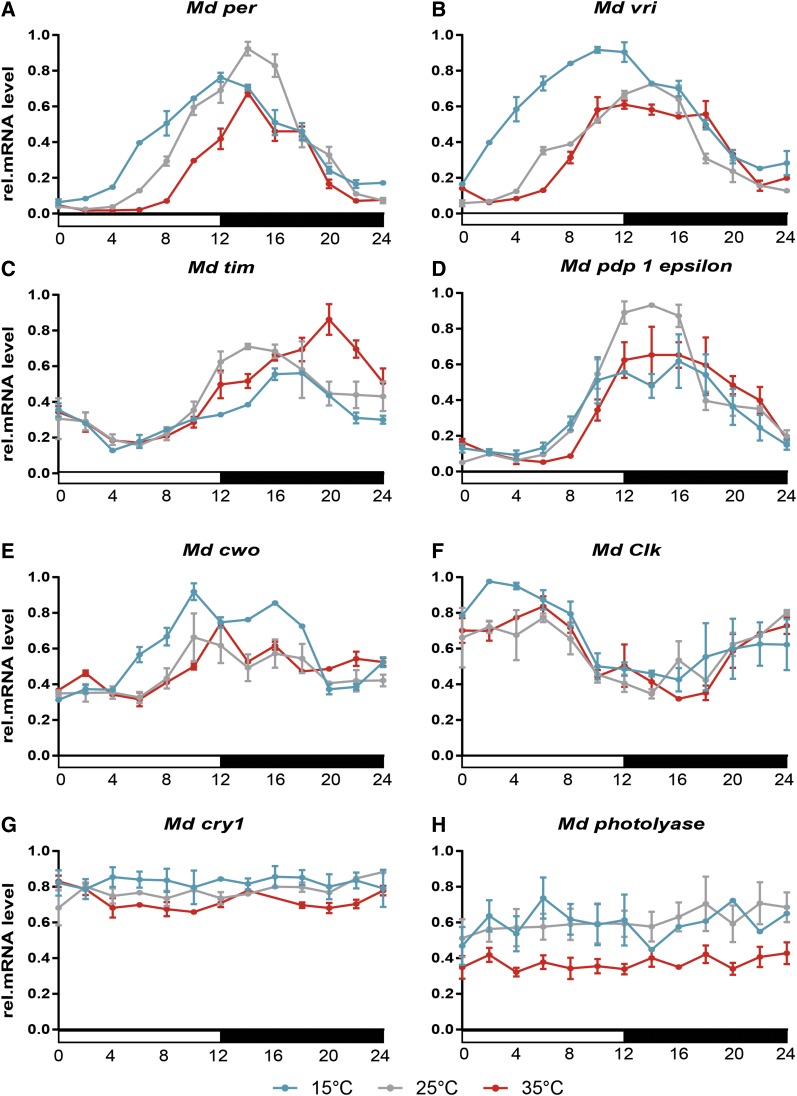
Relative mRNA level of clock gene expression in *M. domestica* heads at three different constant temperatures (15° – blue line, 25° – gray line, and 35° – red line) under the short photoperiod (LD 12:12). The average of three independent biological replicates is shown for each temperature ±SD. (A) *period*, (B) *vrille*, (C) *timeless*, (D) *Par domain protein 1 epsilon*, (E) *clockwork orange*, (F) *Clock*, (G) *cryptochrome1*, and (H) *photolyase*. See *Results* section and Table S3 (A–H) for statistical analysis.

A previous study identified the peak of *per* abundance at Zeitgeber time (ZT) 16 in houseflies kept at 25° (Codd *et al.* 2007). Our data, relying on more detailed 2 hr resolutions, indicated the mRNA maximum at ZT 14 (Figure 5A). The expression rise and its peak were advanced by ∼2 hr at 15°. Consistently, *Md_per* accumulation started with ∼2 hr delay at 35°; however, the peak and descending arm was identical for both 35 and 25° (Figure 5A).

A similar trend was observed in *Md_vri*, where exposure to 15° resulted in 5–6 hr advancements in the mRNA expression profile (Figure 5B). In contrast to *Md_per*, no difference was observed in *Md_vri* expression at 25° Compared to 35° (Figure 5B gray *vs.* magenta). A comparable trend to *Md_vri* expression, a phase advancement at 15°, and no difference between expressions at 25 and 35° were found in *Md_cwo* (Figure 5E) and *Md_Clk* (Figure 5F). The main difference was in the phase of expression: *Md_Clk* started to accumulate during the scotophase and peaked early in the photophase, particularly at 15° (Figure 5F), whereas the *Md_cwo* peak lies at the end of the photophase (Figure 5E) similar to *Md_per*, *Md_vri*, and *Md_Pdp1_epsilon_* (Figure 5, A, B, and D).

The expressions of *Md_tim* were influenced differently with changing temperatures. Similar to the *Md_per* maximum, at 25°, mRNA peaked at ZT 14 (Figure 5C). A low temperature of 15° resulted in a phase delay (peak at ZT 16–18) and a slightly lower expression level. Moreover, a high temperature of 35° delayed the phase of the *Md_tim* maximum even more (peak at ZT 20) and the expression level was higher than that observed at 15 or 25° (Figure 5C).

Temperature did not affect the phase of *Md_Pdp1_epsilon_*, which peaked during the early photophase at all three temperatures (Figure 5D). The expression level of *Md_cry* was temperature-independent and noncyclical (Figure 5G), confirming results published previously (Codd *et al.* 2007). Similarly, the expression of *Md_photolyase* was noncyclical (Figure 5H).

### mRNA expression at different temperatures: D. melanogaster

**Figure 6:**
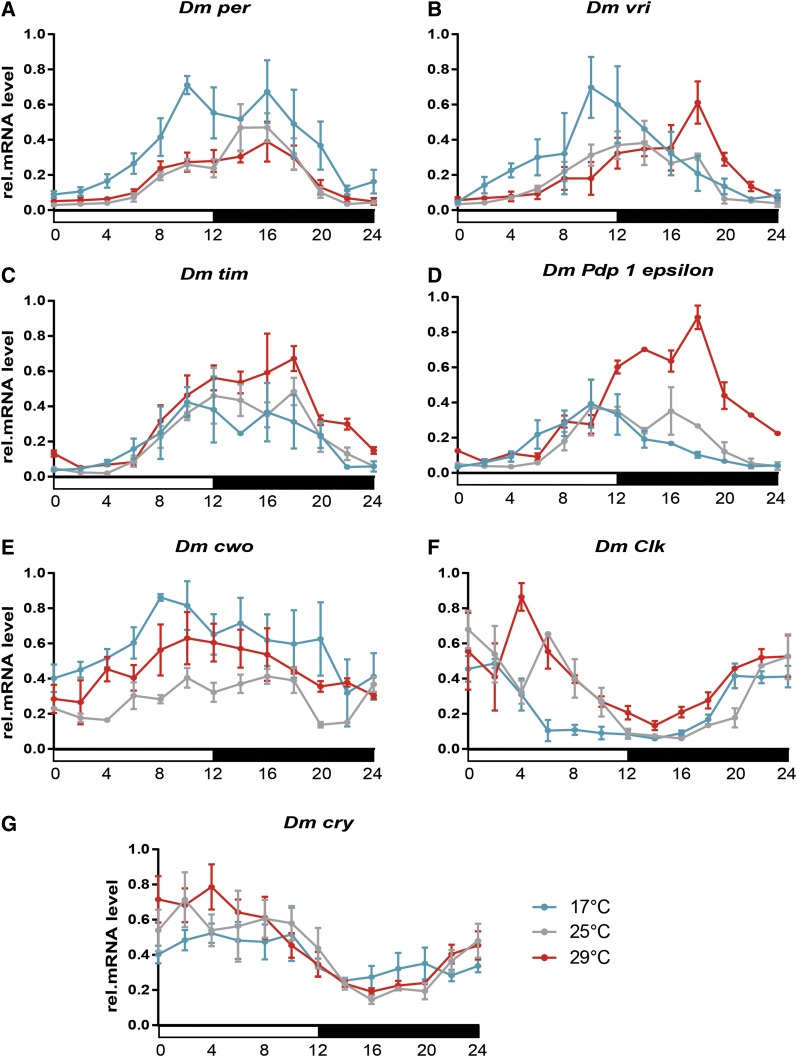
Relative mRNA levels of clock gene expression in *D. melanogaster* heads at three different constant temperatures (17° – blue line, 25° – gray line, and 29° – red line) under the short photoperiod (LD 12:12). The average of three independent biological replicates is shown for each temperature ±SD (A) *period*, (B) *vrille*, (C) *timeless*, (D) *Par domain protein 1 epsilon*, (E) *clockwork orange*, (F) *Clock*, and (G) *cryptochrome1*. See *Results* section and Table S4 (A–G) for statistical analysis.

To compare if similar trends were conserved in *D. melanogaster*, expression profiles at low (18°), ambient (25°), and high (29°) temperatures were defined with a 2 hr resolution. The *Dm_per* peak was phase advanced to ZT 10 at low temperatures (Figure 6A), whereas mRNA reached its maximum at ZT 14–16 at both ambient and high temperatures. Similarly, the *Dm_vri* peak was advanced and delayed at 18 and 29°, respectively (Figure 6B). Similar to *Musca*, the accumulation of *Dm_tim* started identically at all three temperatures (Figure 6C). The only differences, bordering on a statistically significant value (see Table S5), were observed later in the middle of the scotophase, where higher temperatures were associated with the higher *Dm_tim* levels in contrast to the low temperature samples (Figure 6C). The *Dm_Pdp1_epsilon_* was influenced by temperature in a different manner from that of *Md_Pdp1_epsilon_* (Figure 5D). The high temperature resulted in higher *Dm_Pdp1_epsilon_* levels that peaked at ZT 18, ∼8 hr later than the low temperature maximum (Figure 6D).

### Mdpi11 and Mdpi12 splicing

**Figure 7:**
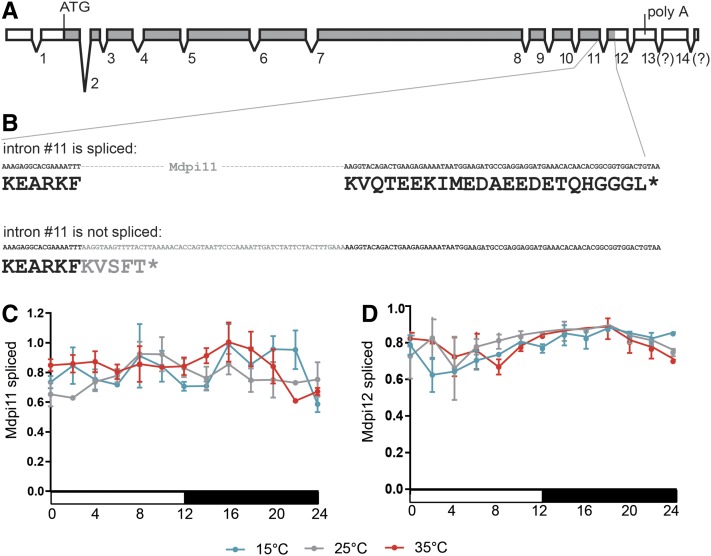
(A) Structure of *M. domestica per* gene with exons (rectangles) and introns (lines). Gray fill corresponds to coding sequence; position of the start codon highlighted (ATG) and stop codon (*… stop codon) are shown. Although the genome annotation predicts 14 introns within the *per* gene, our 3ʹ RACE identified the poly A tail (poly A) in mRNAs corresponding to the 13th exon. (B) Details of intron 11 (in gray color) and surrounding exons with corresponding protein sequence shown below DNA sequence. Both intron Mdpi11 (C) and Mdpi12 (D) are spliced effectively at all three tested temperatures.

Two PER-immunoreactive bands were identified in the head extracts, whereas only a single band had previously been recognized in the thorax (figure 4 in Codd *et al.* 2007). We failed to identify any introns in the 3ʹ UTR of the *Musca* *per* gene corresponding to *dmpi8* (Majercak *et al.* 1999) either by the *in silico* search in the published *Musca* genome (Scott *et al.* 2014), or using a PCR-based approach. Nevertheless, the alternative retention of *mdpi11*, the last intron within the coding sequence, would result in the PER protein being shorter by 18 amino acids (Figure 7, A and B). Therefore, we measured the expression of both transcript variants using a splice isoform-specific primer combination. Clearly, the expression of *mdpi11* was not affected by temperature (Figure 7C). Similarly, temperature had no influence on splicing of the last intron, *mdpi12*, which is localized in 3ʹ UTR (Figure 7D).

### timeless splicing in Musca

**Figure 8:**
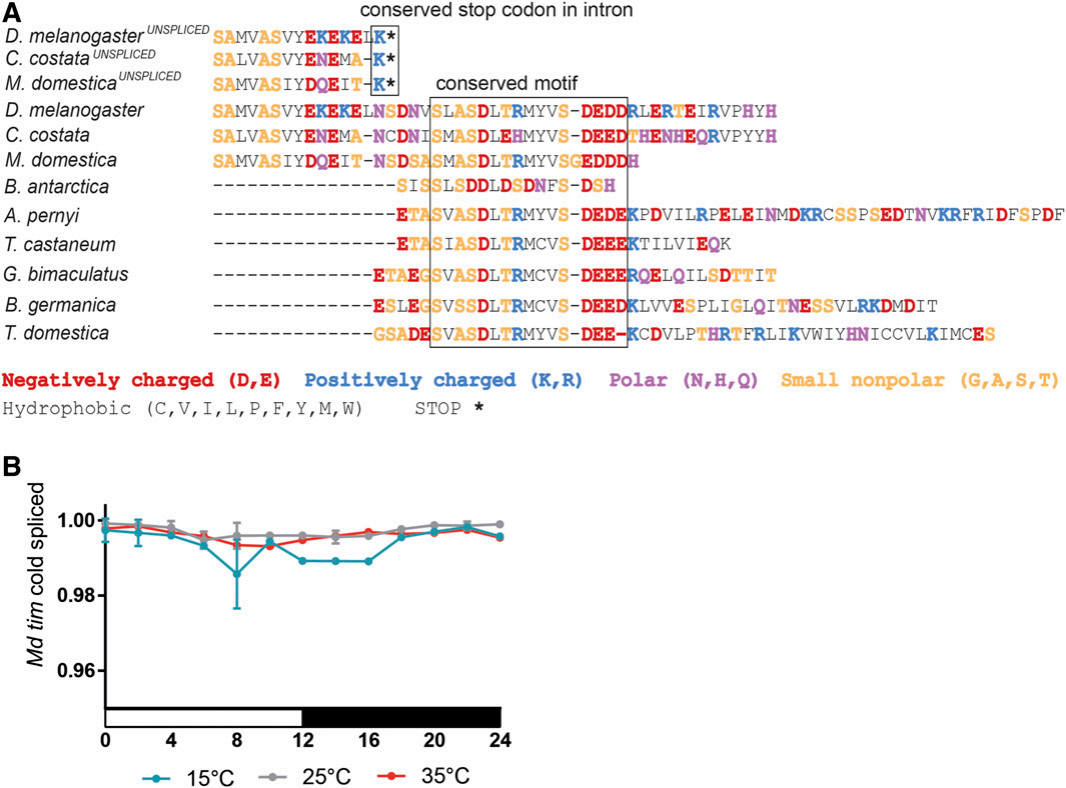
(A) Details of C terminal region alignment of TIM proteins with a highlighted conserved motif in all insect species and conserved stop codon resulting from putative unspliced transcripts in *D. melanogaster*, *C. costata*, and *M. domestica*. (B) This last *tim* intron is spliced effectively in *M. domestica* at all three tested temperatures.

Interspecific comparisons of TIM proteins across all insect species identified 14–15 conserved amino acid motifs containing 3–4 acidic residues near the C terminus (Figure 8A), whereas its preceding sequences were highly variable between various species. Low temperatures result in the unspliced last intron of *D. melanogaster tim* containing an earlier stop codon completely removing this conserved TIM motif (Boothroyd *et al.* 2007; Montelli *et al.* 2015). A comparison of the genomic sequence identified the existence of identical intronic premature stop codons in *C. costata* and *M. domestica* (Figure 8A). Therefore, we explored the splicing frequency of the last intron by measuring the levels of both isoforms: the spliced transcript, resulting in a full protein with a conserved motif, and the nonspliced isoform, resulting in a shorter protein, called TIM^UNSPLICED^. Low temperatures resulted in a ∼1% reduction in the splicing efficiency of *Md_tim* (Figure 8A). Although this difference is statistically significant, low temperatures have a minimal effect when compared to the ∼10-fold splicing difference in the homologous *tim* intron of *D. melanogaster* (Montelli *et al.* 2015).

### Promoter comparison

**Figure 9:**
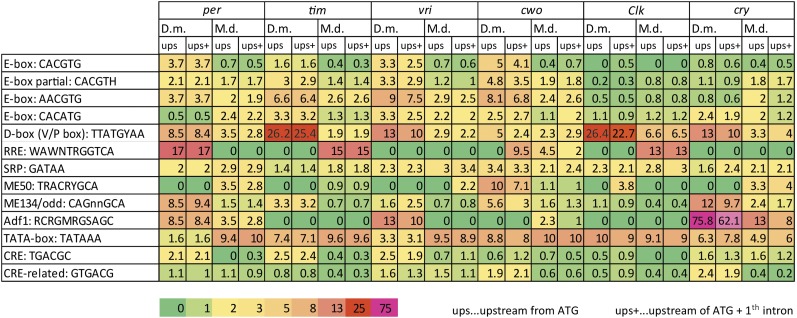
Relative abundance of *cis*-regulatory motifs in promoters of *D. melanogaster* and *M. domestica*. The values indicate the actual occurrence of a particular *cis*-motif presented as a fold change when 1 is the expected frequency of a particular motif in a random DNA sequence. Heat map was used for easier orientation in the table. Two regions of the genes were analyzed: the region upstream of ATG (ups) and the upstream region with the first intron (ups+). The actual position and distribution of selected *cis*-motifs in DNA is shown in Figure 10 and Figure 11 and full maps are presented for *cwo* genes in Figure S3.

Having the *M. domestica* genome in hand (Scott *et al.* 2014), we explored the promoter regions for the presence of putative *cis*-regulatory sequences and compared them with promoters in *D. melanogaster*. No experimental data defined the length of the promoter regions in *M. domestica*, and only some *D. melanogaster* circadian genes were functionally explored using systematic promoter truncation experiments. Therefore, we analyzed the DNA region upstream of the start codon (“ups” in Figure 9) and also separately analyzed regions including the first intron after the start codon (“ups+” in Figure 9).

In general, *M. domestica* genes were longer than *D. melanogaster* homologs, often with larger intronic sequences. The frequency of E-box-related motifs (CACGTG, CACGTH, AACGTG) was lower in *M. domestica* than in *D. melanogaster* for the *per*, *tim*, *vrille*, and *cwo* promoters (Figure 9). The frequency of the canonical E-box (CACGTG) was even lower than expected for random occurrences of this motif in *Md*_*per*, *Md*_*tim*, *Md*_*vri*, and *Md*_*cwo*.

**Figure 10:**
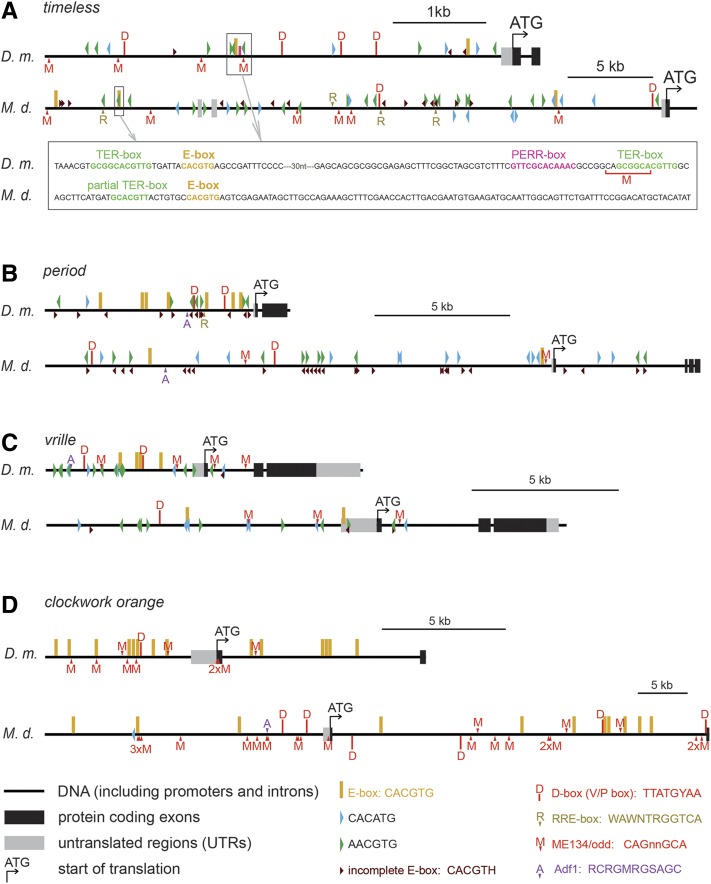
Schematic depictions of *D. melanogaster* (*D.m*.) and *M. domestica* (*M.d*.) promoters with highlighted positions of putative *cis*-regulatory motifs in (A) *timeless*, (B) *period*, (C) *vrille*, and (D) *clockwork orange*. In the case of *cwo* (D), E-box and related *cis*-regulatory motifs were omitted for picture clarity. For the complete map see Figure S3. Note different scales (shown on the right for each gene).

**Figure 11:**
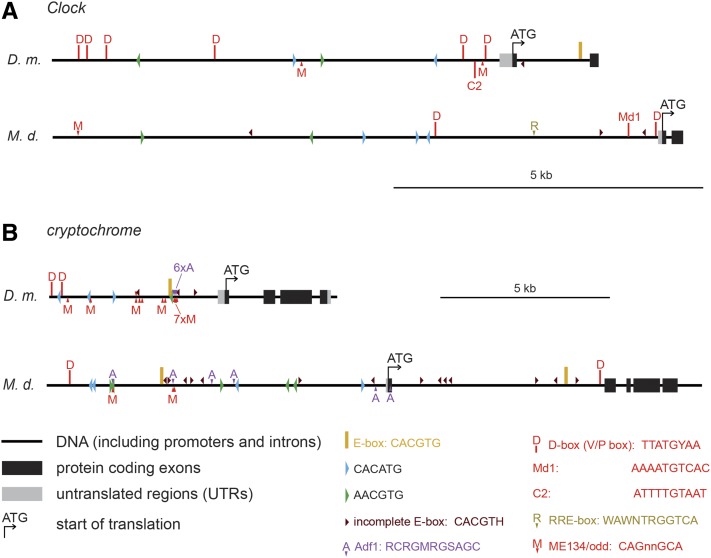
Schematic depictions of *D. melanogaster* (*D.m*.) and *M. domestica* (*M.d*.) promoters with highlighted positions of putative *cis*-regulatory motifs in (A) *Clock*, and (B) *cryptochrome*. C2 motif (Cyran *et al.* 2003) and Md1 motif are similar to D-box. Note different scales (shown on the right for each gene).

Remarkably, frequent occurrences of the D-box (known also as the V/P box) were found in *Dm_tim* (26-fold), *Dm_Clk* (26 fold), *Dm_cry* (13-fold), and *Dm_vri* (13-fold). In *M. domestica*, the D-box motif was less abundant (*i.e.*, 6.6-fold in *Md_Clk*, 3.3-fold in *Md_cry*). An RRE-element (Harding and Lazar 1993) was found in *Dm_per* (17-fold), *Md_tim* (15-fold), and *Md_Clk* (13-fold). The Adf1 motif (Meireles-Filho *et al.* 2014) was strikingly abundant in *Dm_cry* (76-fold) and also frequent in *Dm_per* (8.5-fold), *Dm_vri* (13-fold), and *Md_cry* (13-fold) (see Figure 9 for full comparison of *cis*-motif frequency, Figure 10 for position of motifs in *tim*, *per*, *vri* and *cwo* promoters, and Figure 11 for *Clk* and *cry* promoters).

## Discussion

The expression of circadian genes was explored in many insect species. In general, expression patterns often differ among tissues (Beaver *et al.* 2003; Iwai *et al.* 2006; Tomioka *et al.* 2012; Damulewicz *et al.* 2015), is affected by the reproductive/diapause status of the animal (Dolezel *et al.* 2008; Kostal *et al.* 2008; Bajgar *et al.* 2013a,b), and depends on the animals’ age and access to nutrition (Dolezel *et al.* 2007; Rakshit *et al.* 2012).

The temporal expression of circadian genes also remarkably differs between species. The strong cyclical expression characteristic of several circadian genes in *Drosophila* is not the rule for all insect species. For instance, the expression of circadian clock gene homologs *per* and *Clk* show no cyclical changes either in heads of the linden bug, *P. apterus* (Syrova *et al.* 2003), or even in dissected brains (J. Kotwica-Rolinska and D. Dolezel, unpublished data). More importantly, expression changes differ between different brain regions of *D. melanogaster*, including the prominent amplitude of many transcripts in PDF cells (Kula-Eversole *et al.* 2010). Notably, the expression in compound eyes contributes remarkably to whole head mRNA levels of several circadian genes. Indeed, the identification of the *cryptochrome^b^* mutation in a luciferase-reporter screen (Stanewsky *et al.* 1998) was made possible by this strong expression in the eye. Therefore, correlating expression patterns in the whole head with the locomotor activity profile is only an approximation and overlooks cell-specific expressions. However, the lack of suitable antibodies recognizing *Musca* circadian genes prevents rigorous analysis with anatomical resolution. At the same time, expression profiles on entire *D. melanogaster* heads were used extensively; thus, interspecific comparisons with solid data are feasible.

**Figure 12:**
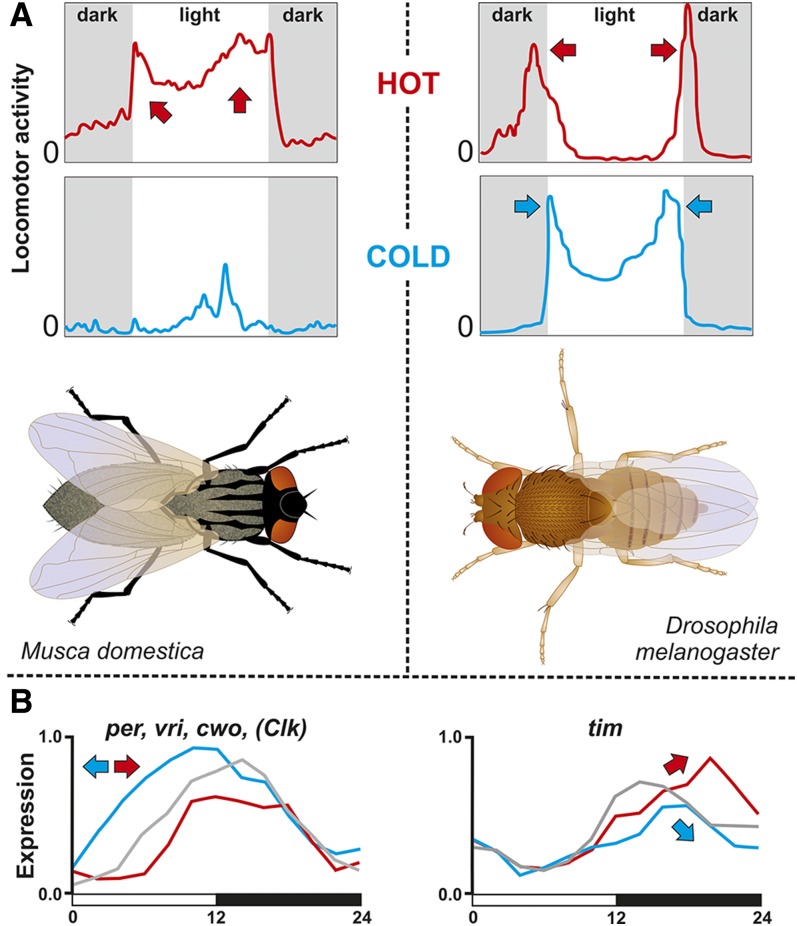
Summary of response to temperature in (A) *M. domestica* (left) and *D. melanogaster* (right). Although the locomotor activity pattern is different, both species display bimodal activity patterns at hot temperatures. Under cold temperatures, *M. domestica* reduces its activity to a single peak in the center of the photophase, whereas the activity of *D. melanogaster* is still bimodal; however, the position of both peaks moves toward the photophase. (B) Low temperatures result in the phase advancement of *per*, *vri*, *cwo*, and *Clk* expression in both species (left panel). In *Musca*, both hot and cold temperatures result in the amplitude change of *tim* expression and also in phase shift (right panel).

In this study, we addressed the impact of temperature on the expression of circadian genes in *Musca* compared with that of *D. melanogaster* homologs. Despite certain differences between these two species, a few general patterns were observed (Figure 12). Low temperatures resulted in the phase advancement of *period*, *vrille*, and *cwo* in both species (Figure 5 and Figure 6). However, the phase of *Pdp1_epsilon_* expression was not affected in *Musca*, whereas the expression of *Dm_Pdp1_epsilon_* was regulated similarly to *Dm_tim* (see below). Furthermore, the expression of *Md_tim* was clearly delayed by both high and low temperatures in *M. domestica* and temperature affected also the amplitude of *Md_tim*. However, *Dm_tim* differed only in the amplitude with no phase shift. Our *Dm_tim* expression is in agreement with results observed previously (Kidd *et al.* 2015), where *tim* mRNA and TIM protein expression served as a case support for a so-called “pathway” model of temperature compensation (Kidd *et al.* 2015). This model expects that “the concentration of the protein and mRNA components of the clock should scale in a simple fashion with temperature. In particular, the overall amplitude or average value of an oscillation in any given component can change with temperature, but the shape of the oscillation and the phase relationships between different oscillating components should remain approximately the same at any temperature…” (Kidd *et al.* 2015). Although our experiment did not address temperature compensation directly and data were obtained under an LD regime only, clear intraspecific difference in *tim* expression suggests further validation of the proposed pathway model. This caution is further strengthened by the more complex impact of temperature on expression of additional circadian clock genes, where phase was also affected.

The transcription of *per*, *tim*, *Pdp1_epsilon_*, *vri*, and *cwo* requires the CLK-CYC heterodimer (Cyran *et al.* 2003); therefore, it was surprising to see different expression patterns in these genes. One possible explanation is the different temperature-dependent transcription. Indeed, CLK and CYC tissue-specific expression of *Drosophila* is enhanced by the transcription factors ODD PAIRED and SERPENT (Meireles-Filho *et al.* 2014). Similar alterations and redirections might contribute to the temperature-adjustment of circadian gene expression. Alternatively, posttranscriptional regulation might contribute to altered mRNA stability and, therefore, affect its accumulation. Indeed, three circadian genes, *vri*, *cwo*, and *Clk*, are regulated by microRNAs in *D. melanogaster* (Kadener *et al.* 2009). Most likely, the combination of both phenomena contributes to the temperature compensation mechanism.

The most striking difference was found in *cry* expression, which is flat in *Md_cry* (Codd *et al.* 2007, and Figure 5), whereas *Dm_cry* expression is cyclical with a peak during the photophase (Emery *et al.* 1998, and Figure 6). Although some weak phenotypes are found at nonambient temperatures for *cry*-null mutants (Dolezelova *et al.* 2007), *cry* is not a key component of the circadian clock under constant dark conditions. Instead, the CRY protein serves as a sensitive light receptor for the entrainment of the clock (Emery *et al.* 1998, 2000).

To further shed light on a possible mechanistic explanation of transcriptional regulation, extensive comparison of *cis*-regulatory motifs was performed. We were hoping to recognize some clear pattern that would define further functional tests. A few findings are worthy of attention: It is tempting to speculate that different expression of *tim* between these two fly species results from remarkably different frequency of D-box motif in *tim* promoters (4× D-box/5 kb in *D. melanogaster* *vs.* 2× D-box/30 kb in *M. domestica*). PDP1_epsilon_ and VRI, two basic Leucine Zipper Domain (bZIP) circadian proteins, are known to bind to D-box motif in *D. melanogaster* (Cyran *et al.* 2003).

Another gene, with remarkably different expression between compared species was *cry*, which was cycling in *D. melanogaster*, but flat in *M. domestica*. While the frequency of E-box motifs was comparable in *cry* promoters of both species, *D. melanogaster cry* promoter was enriched for Adf1 motif and for D-box. Although this analysis was purely computational and hence identified only putative binding motifs, rigorous functional tests are easy in *D. melanogaster* and now even possible in *Musca* thanks to gene editing technology (Sharma *et al.* 2017).

Our locomotor activity experiments identified the clear impact of temperature on the activity distribution pattern in *M. domestica*. While at low temperatures houseflies showed a single activity peak located at the middle of the photophase, their activity was distributed throughout the entire photophase under ambient and high temperatures. Although one could expect that houseflies exposed to high temperatures will try to avoid dry midday and afternoon, minimal differences were observed between ambient and high temperatures. Notably, houseflies successfully colonized remarkably different environments including dry and humid regions. Since each experimental tube contained a water reservoir, we expect that the humidity was relatively high. It would be interesting to explore behavior under more natural conditions (see also text below).

The activity profile of males at ambient and high temperatures contained mild troughs between the light on and off peaks, exhibiting similarities with morning and evening activity peaks of *D. melanogaster*. It is believed that lower midday activity, the “siesta,” is an adaptation protecting *D. melanogaster* from dry environments experienced at noon. However, this behavior was observed under constant temperatures in laboratory experiments. Locomotor activities obtained from *D. melanogaster* recorded under natural conditions, with temperature cycles during the day (together with the light intensity), revealed more complex patterns with an additional noon peak (Vanin *et al.* 2012; Green *et al.* 2015). Despite these discrepancies, the daily activity pattern is clearly affected by either constant or cyclical temperature profiles in *D. melanogaster*.

Temperature-dependent splicing of the last *per* intron (*Dmpi8*) is established as the key factor influencing the timing of morning and evening activity peaks (Majercak *et al.* 1999, 2004; Cao and Edery 2017). Therefore, we searched for a homolog of this 3ʹ UTR intron in *Musca*. However, the expression analysis of both introns localized at the 3ʹ did not reveal any temperature-influenced splicing, suggesting a different molecular mechanism may be involved in *M. domestica*. This mechanism might include the splicing of introns localized in the open reading frame. Alternatively, the expression profile of additional circadian genes might be important. The latter explanation is supported by conserved temperature-dependent expression trends between *D. melanogaster* and *M. domestica*.

In *D. melanogaster*, low temperatures result in the *tim*^UNSPLICED^ isoform removing the last 34 amino acid of the TIM protein including the conserved 15 amino acid motif. The importance of this motif is suggested by its presence in *Thermobia domestica*, an insect species that shared a common ancestor with *Drosophila* >400 MYA (Misof *et al.* 2014). Surprisingly, the motif is highly modified in *Belgica antarctica*, a dipteran insect living in extreme and generally cold environments (Kelley *et al.* 2014). Nevertheless, stop codon positions are conserved in *tim* introns of *D. melanogaster*, *Chymomyza costata*, and *M. domestica*. However, only a few percent reductions in splicing were found at 15° in *M. domestica*. Perhaps even lower temperatures are required for this temperate fly to change its splicing pattern dramatically.

Functional experiments addressing the role of particular genes in *Musca* are needed to fully define underlying biological mechanisms. Alternatively, further comparative studies might point to conserved features across taxa. Interestingly, bimodal activity was found even in distantly related insects, for example in Lepidoptera where the flight activity of *Ephestia kuehniella* is restricted to early and late night peaks (Zavodska *et al.* 2012; Kobelkova *et al.* 2015). It would be interesting to see how this nocturnal insect changes its activity pattern at different temperatures, if the expression of circadian genes is affected by the temperature, and how. Similar comparative experiments might identify general patterns, which need to be verified functionally. These reverse genetics experiments are technically demanding in nonmodel insects, yet current gene editing tools are already reaching chronobiology (Markert *et al.* 2016; Sharma *et al.* 2017).

## Supplementary Material

Supplemental material is available online at www.g3journal.org/lookup/suppl/doi:10.1534/g3.117.042374/-/DC1.
