# Supplementary material for: Daily Activity of the Housefly, Musca domestica, Is Influenced by Temperature Independent of 3′ UTR period Gene Splicing
Source: G3 (Bethesda). 2017 Jun 15;7(8):2637–49. doi: 10.1534/g3.117.042374 (PMC5555469; doi:10.1534/g3.117.042374)
Supplement: Supplementary file 1 [file 2637FigureS1.docx]

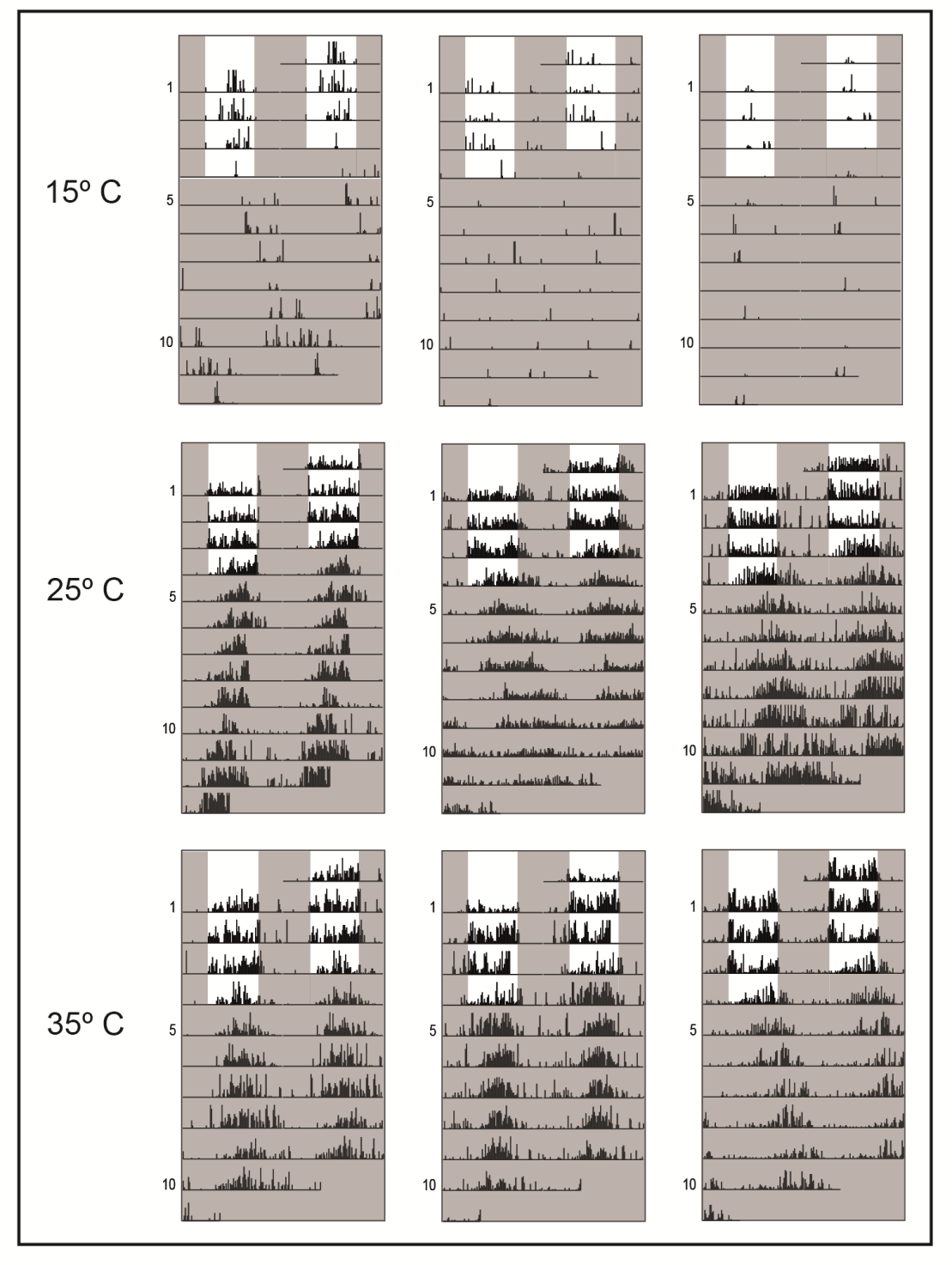


**Figure S1** Examples of double-plotted actograms of flies entrained to a photoperiod of LD 8:16 for 3 d followed by constant dark conditions (first day in DD marked by an arrow). In the first line are actograms of individual flies kept under low temperatures (15 ℃). The second line illustrates actograms of flies placed at ambient temperature (25 ℃) -neither males, nor females had a free running period shorter than 23 h. Actograms of flies placed under high temperatures (35 ℃) are visualized in the last line.
