# Supplementary material for: Daily Activity of the Housefly, Musca domestica, Is Influenced by Temperature Independent of 3′ UTR period Gene Splicing
Source: G3 (Bethesda). 2017 Jun 15;7(8):2637–49. doi: 10.1534/g3.117.042374 (PMC5555469; doi:10.1534/g3.117.042374)
Supplement: Supplementary file 2 [file 2637FigureS2.docx]

**
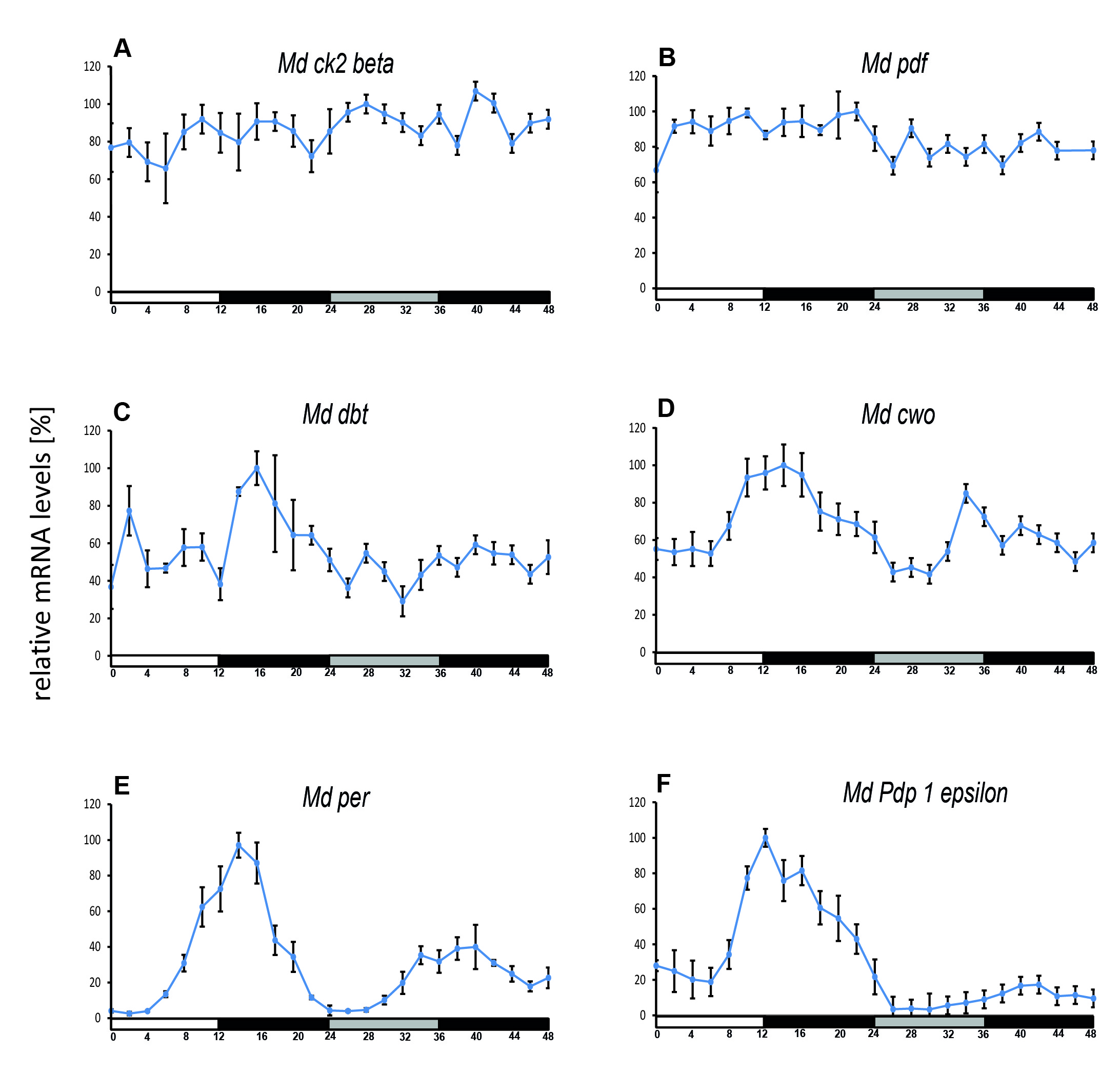
**

**Figure** S2 Relative mRNA levels of (A) *casein kinase 2 beta,* (B) *pigment dispersing factor,* (C) *doubletime (casein kinase 1 epsilon),* (D) *clockwork orange,* (E) *period* and (F) *par domain protein 1 epsilon*). The average of three independent biological replicates is shown, +/- SD.
