# Supplementary material for: Daily Activity of the Housefly, Musca domestica, Is Influenced by Temperature Independent of 3′ UTR period Gene Splicing
Source: G3 (Bethesda). 2017 Jun 15;7(8):2637–49. doi: 10.1534/g3.117.042374 (PMC5555469; doi:10.1534/g3.117.042374)
Supplement: Supplementary file 3 [file 2637FigureS3.docx]

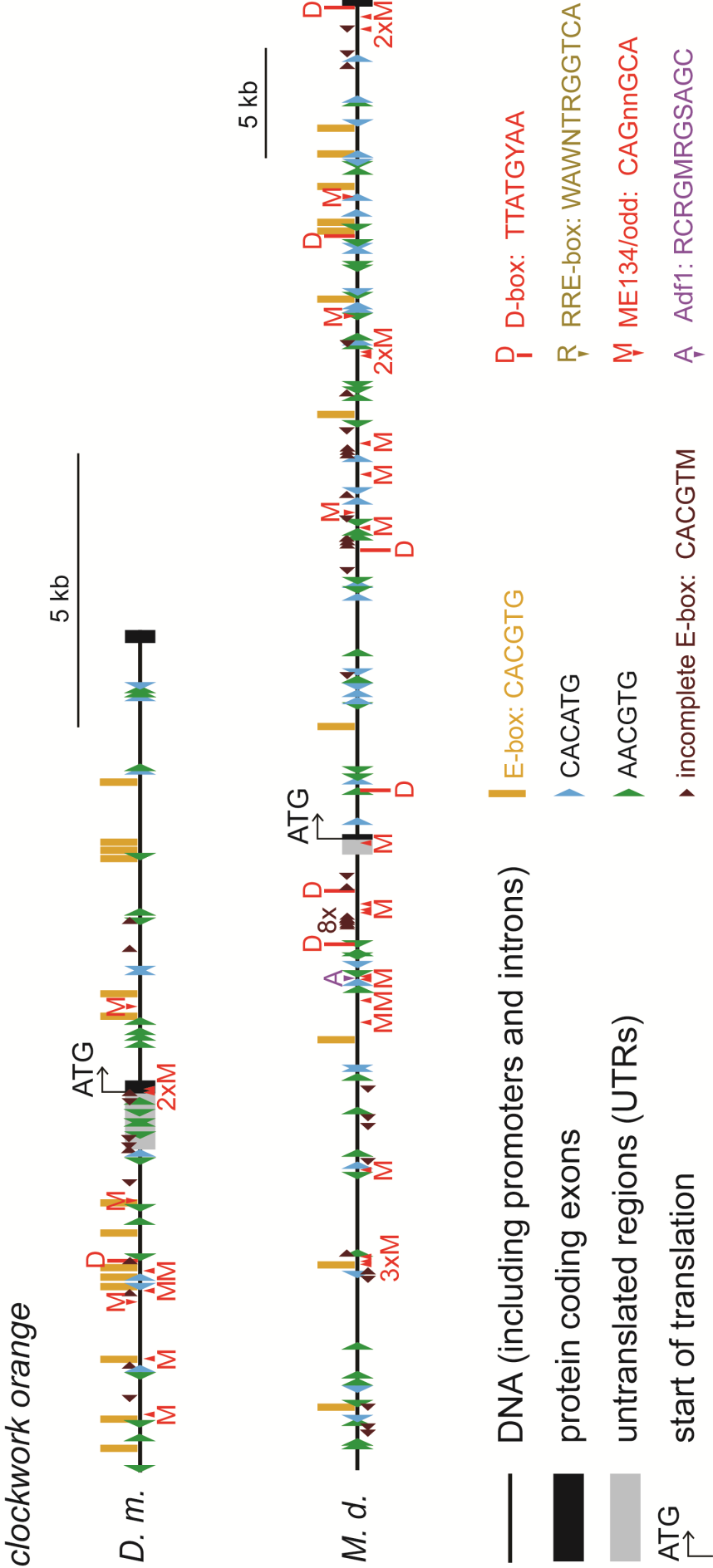


**Figure** S3 Schematic depictions of *D. melanogaster* (*D.m.*) and *M. domestica* (*M.d.*) *clockwork orange* promoters with highlighted positions of putative cis-regulatory motifs. Note different scales (shown on the right for each gene).
