# Supplementary material for: Daily Activity of the Housefly, Musca domestica, Is Influenced by Temperature Independent of 3′ UTR period Gene Splicing
Source: G3 (Bethesda). 2017 Jun 15;7(8):2637–49. doi: 10.1534/g3.117.042374 (PMC5555469; doi:10.1534/g3.117.042374)
Supplement: Supplementary file 4 [file 2637TableS1.docx]

**Table S1** Primers for RT-PCR expression analysis of *Musca domestica* circadian genes.

| **transcript** | **primer** | **Primer sequence^1^** | **Product size (bp)** |
| --- | --- | --- | --- |
|  |  |  |  |
| *rp49* | rp49 fw | 5´GTTATGCCAAATTGTCG**^**CACA 3´ | 123 |
|  | rp49 rev | 5´GGCGGGTACGTTTGTTGG 3´ |  |
| *clock work orange* | cwo fw | 5´TCTACAGAGTGAGTGCATGCAGAAGG 3´ | 166 |
|  | cwo rev | 5´ACAGTCAT**^**TTTTCATAACCTCTTC 3´ |  |
| *pigment dispersing hormone* | pdh fw | 5´GAGCAGTACTTTGATAAAC**^**AGCTG 3´ | 254 |
|  | pdh rev | 5´GTCTTTTATCTTCTTCTTATCCATAGG 3´ |  |
| *casein kinase 2 beta* | ck2beta fw | 5´GTGGCATAGCACAAATGATTG 3´ | 291 |
|  | ck2beta rev | 5´AAAGT**^**CTTGGAACAAATTGATTAG 3´ |  |
| *par domain protein 1 epsilon* | pdp fw | 5´CGAATAATACAGTGCAGCAGGTGG 3´ | 128 |
|  | pdp rev | 5´GCTATTCGATGTGGATTTACC**^**TGA3´ |  |
| *period ^2^* | per fw1 | 5´ACGAAAACACTCTTAAG**^**CCCAA 3´ | 118 |
|  | per rev1 | 5´TTTGCTGTTGTCGTTCTCCTG 3´ |  |
| *period varA* | per fw2 | 5´GCTCCATCGGATTCTTCTAAAAAG 3´ | 319 |
|  | per rev2 | 5´TTCTCTTCAGTCTGTAC**^**CTTAAAT 3´ |  |
| *period varB* | per fw1 | 5´ACGAAAACACTCTTAAG**^**CCCAA 3´ | 235 |
|  | per rev3 | 5´ TTCTCTTCAGTCTGTACCTTTTTC 3´ |  |
| *timeless^2^* | tim fw1 | 5´ TGTTGCTCTTGATACTGGATAGTG 3´ | 118 |
|  | tim r1 | 5´AGCAGGATGCCATAGAAGTG 3´ |  |
| *casein kinase 1 epsilon* ^3^ | dbt fw | 5´ACTTCTCGGACCCTCACTCG 3´ | 164 |
|  | dbt rev | 5´GCCCATTAGGAAATTGTCTGG 3´ |  |
| *vrille* ^2^ | vri fw | 5´AATGAGGCCACAAATG^TTCAC 3´ | 156 |
|  | vri rev | 5´ GGCGCTGACCTGCTGTTT 3´ |  |

^1^ intron position is marked with a caret (**^**)

^2^ primers used previously by Codd *et al.* 2007

^3^ intron not found – RNA treated by DNase
