# Supplementary material for: Daily Activity of the Housefly, Musca domestica, Is Influenced by Temperature Independent of 3′ UTR period Gene Splicing
Source: G3 (Bethesda). 2017 Jun 15;7(8):2637–49. doi: 10.1534/g3.117.042374 (PMC5555469; doi:10.1534/g3.117.042374)
Supplement: Supplementary file 5 [file 2637TableS2.docx]

**Table S2** – annotation symbols of *Drosophila* circadian genes

| *Dm period* | CG2647 |
| --- | --- |
| *Dm timeless* | CG3234 |
| *Dm vrille* | CG14029 |
| *Dm Pdp1* | CG17888 |
| *Dm clockwork orange* | CG17100 |
| *Dm Clock* | CG7391 |
| *Dm cryptochrome* | CG3772 |
| *Md period* | MDOA015102 |
| *Md timeless* | MDOA011126 |
| *Md vrille* | EU075345 |
| *Md clockwork orange* | MDOA001233 |
| *Md Clock* | MDOA010622 |
| *Md cryptochrome* | MDOA009806 |
| *Md photolyase* | XM_011293895.2 |
