# Supplementary material for: Daily Activity of the Housefly, Musca domestica, Is Influenced by Temperature Independent of 3′ UTR period Gene Splicing
Source: G3 (Bethesda). 2017 Jun 15;7(8):2637–49. doi: 10.1534/g3.117.042374 (PMC5555469; doi:10.1534/g3.117.042374)
Supplement: Supplementary file 6 [file 2637TableS3.docx]

**Table S3.** Statistical comparison of locomotor activity levels by two-way ANOVA (Graphpad Prims). ****p<0.0001, ***p<0.001, **p<0.01, *p<0.05, ns… non-significant. The table accompanies Figure 3 in the manuscript.

| 15M 12:12 vs. 15M 16:8 | 22.77 | -182.8 to 228.3 | No | ns |
| --- | --- | --- | --- | --- |
| 15M 12:12 vs. 25M 12:12 | -526.1 | -742.3 to -309.8 | Yes | **** |
| 15M 12:12 vs. 25M 16:8 | -704 | -911.1 to -496.9 | Yes | **** |
| 15M 12:12 vs. 35M 12:12 | -890.9 | -1113 to -669.0 | Yes | **** |
| 15M 12:12 vs. 35M 16:8 | -1177 | -1391 to -963.5 | Yes | **** |
| 15M 12:12 vs. 15F 12:12 | 23.09 | -268.4 to 314.6 | No | ns |
| 15M 12:12 vs. 15F 16:8 | 30.64 | -186.2 to 247.4 | No | ns |
| 15M 12:12 vs. 25F 12:12 | -271.5 | -506.2 to -36.85 | Yes | ** |
| 15M 12:12 vs. 25F 16:8 | -416.9 | -621.0 to -212.7 | Yes | **** |
| 15M 12:12 vs. 35F 12:12 | -548.2 | -770.5 to -326.0 | Yes | **** |
| 15M 12:12 vs. 35F 16:8 | -830.4 | -1034 to -626.9 | Yes | **** |
| 15M16:8 vs. 25M 12:12 | -548.8 | -732.4 to -365.2 | Yes | **** |
| 15M16:8 vs. 25M 16:8 | -726.8 | -899.5 to -554.1 | Yes | **** |
| 15M16:8 vs. 35M 12:12 | -913.7 | -1104 to -723.4 | Yes | **** |
| 15M16:8 vs. 35M 16:8 | -1200 | -1381 to -1019 | Yes | **** |
| 15M16:8 vs. 15F 12:12 | 0.3159 | -267.9 to 268.5 | No | ns |
| 15M16:8 vs. 15F 16:8 | 7.87 | -176.4 to 192.1 | No | ns |
| 15M16:8 vs. 25F 12:12 | -294.3 | -499.3 to -89.33 | Yes | *** |
| 15M16:8 vs. 25F 16:8 | -439.6 | -608.8 to -270.5 | Yes | **** |
| 15M16:8 vs. 35F 12:12 | -571 | -761.7 to -380.4 | Yes | **** |
| 15M16:8 vs. 35F 16:8 | -853.2 | -1022 to -684.7 | Yes | **** |
| 25M12:12 vs. 25M 16:8 | -177.9 | -363.2 to 7.392 | No | ns |
| 25M12:12 vs. 35M 12:12 | -364.8 | -566.6 to -163.1 | Yes | **** |
| 25M12:12 vs. 35M 16:8 | -651.1 | -843.8 to -458.5 | Yes | **** |
| 25M12:12 vs. 15F 12:12 | 549.2 | 272.7 to 825.6 | Yes | **** |
| 25M12:12 vs. 15F 16:8 | 556.7 | 360.6 to 752.8 | Yes | **** |
| 25M12:12 vs. 25F 12:12 | 254.5 | 38.83 to 470.2 | Yes | ** |
| 25M12:12 vs. 25F 16:8 | 109.2 | -72.80 to 291.2 | No | ns |
| 25M12:12 vs. 35F 12:12 | -22.17 | -224.3 to 180.0 | No | ns |
| 25M12:12 vs. 35F 16:8 | -304.3 | -485.7 to -123.0 | Yes | **** |
| 25M16:8 vs. 35M 12:12 | -186.9 | -378.8 to 5.026 | No | ns |
| 25M16:8 vs. 35M 16:8 | -473.2 | -655.6 to -290.9 | Yes | **** |
| 25M16:8 vs. 15F 12:12 | 727.1 | 457.7 to 996.5 | Yes | **** |
| 25M16:8 vs. 15F 16:8 | 734.6 | 548.7 to 920.6 | Yes | **** |
| 25M16:8 vs. 25F 12:12 | 432.5 | 225.9 to 639.0 | Yes | **** |
| 25M16:8 vs. 25F 16:8 | 287.1 | 116.1 to 458.1 | Yes | **** |
| 25M16:8 vs. 35F 12:12 | 155.8 | -36.54 to 348.1 | No | ns |
| 25M16:8 vs. 35F 16:8 | -126.4 | -296.7 to 43.90 | No | ns |
| 35M12:12 vs. 35M 16:8 | -286.3 | -485.4 to -87.29 | Yes | *** |
| 35M12:12 vs. 15F 12:12 | 914 | 633.0 to 1195 | Yes | **** |
| 35M12:12 vs. 15F 16:8 | 921.5 | 719.2 to 1124 | Yes | **** |
| 35M12:12 vs. 25F 12:12 | 619.3 | 398.0 to 840.7 | Yes | **** |
| 35M12:12 vs. 25F 16:8 | 474 | 285.3 to 662.7 | Yes | **** |
| 35M12:12 vs. 35F 12:12 | 342.6 | 134.5 to 550.8 | Yes | **** |
| 35M12:12 vs. 35F 16:8 | 60.49 | -127.6 to 248.6 | No | ns |
| 35M16:8 vs. 15F 12:12 | 1200 | 925.8 to 1475 | Yes | **** |
| 35M16:8 vs. 15F 16:8 | 1208 | 1015 to 1401 | Yes | **** |
| 35M16:8 vs. 25F 12:12 | 905.7 | 692.5 to 1119 | Yes | **** |
| 35M16:8 vs. 25F 16:8 | 760.3 | 581.4 to 939.3 | Yes | **** |
| 35M16:8 vs. 35F 12:12 | 629 | 429.6 to 828.4 | Yes | **** |
| 35M16:8 vs. 35F 16:8 | 346.8 | 168.5 to 525.1 | Yes | **** |
| 15F12:12 vs. 15F 16:8 | 7.554 | -269.4 to 284.5 | No | ns |
| 15F12:12 vs. 25F 12:12 | -294.6 | -585.8 to -3.510 | Yes | * |
| 15F12:12 vs. 25F 16:8 | -440 | -707.1 to -172.9 | Yes | **** |
| 15F12:12 vs. 35F 12:12 | -571.3 | -852.5 to -290.1 | Yes | **** |
| 15F12:12 vs. 35F 16:8 | -853.5 | -1120 to -586.8 | Yes | **** |
| 15F16:8 vs. 25F 12:12 | -302.2 | -518.4 to -85.93 | Yes | *** |
| 15F16:8 vs. 25F 16:8 | -447.5 | -630.2 to -264.9 | Yes | **** |
| 15F16:8 vs. 35F 12:12 | -578.9 | -781.6 to -376.2 | Yes | **** |
| 15F16:8 vs. 35F 16:8 | -861 | -1043 to -679.1 | Yes | **** |
| 25F12:12 vs. 25F 16:8 | -145.3 | -348.9 to 58.22 | No | ns |
| 25F12:12 vs. 35F 12:12 | -276.7 | -498.4 to -54.97 | Yes | ** |
| 25F12:12 vs. 35F 16:8 | -558.9 | -761.8 to -355.9 | Yes | **** |
| 25F16:8 vs. 35F 12:12 | -131.4 | -320.5 to 57.74 | No | ns |
| 25F16:8 vs. 35F 16:8 | -413.5 | -580.2 to -246.8 | Yes | **** |
| 35F12:12 vs. 35F 16:8 | -282.2 | -470.6 to -93.70 | Yes | **** |
