# Supplementary material for: Daily Activity of the Housefly, Musca domestica, Is Influenced by Temperature Independent of 3′ UTR period Gene Splicing
Source: G3 (Bethesda). 2017 Jun 15;7(8):2637–49. doi: 10.1534/g3.117.042374 (PMC5555469; doi:10.1534/g3.117.042374)
Supplement: Supplementary file 7 [file 2637TableS4.docx]

**Tables S4.** Statistical comparison of expression analysis in *Musca domestica* by two-way ANOVA (Graphpad Prims). ****p<0.0001, ***p<0.001, **p<0.01, *p<0.05, ns… non-significant. The tables accompany Figure 5 in the manuscript.

| Md per | | | | |
| --- | --- | --- | --- | --- |
| Source of Variation | % of total variation | P value | P value summary | Significant? |
| Interaction | 9.847 | < 0.0001 | **** | Yes |
| zeitgeber time | 80.65 | < 0.0001 | **** | Yes |
| temperature | 6.647 | < 0.0001 | **** | Yes |
| Table S3. A |  |  |  |  |
| Md vri | | | | |
| Source of Variation | % of total variation | P value | P value summary | Significant? |
| Interaction | 13.36 | < 0.0001 | **** | Yes |
| zeitgeber time | 65.61 | < 0.0001 | **** | Yes |
| temperature | 17.65 | < 0.0001 | **** | Yes |
| Table S3. B |  |  |  |  |
| Md tim | | | | |
| Source of Variation | % of total variation | P value | P value summary | Significant? |
| Interaction | 16.03 | < 0.0001 | **** | Yes |
| zeitgeber time | 63.76 | < 0.0001 | **** | Yes |
| temperature | 6.225 | < 0.0001 | **** | Yes |
| Table S3. C |  |  |  |  |
| Md Pdp 1 epsilon | | | | |
| Source of Variation | % of total variation | P value | P value summary | Significant? |
| Interaction | 8.982 | 0.4448 | ns | No |
| zeitgeber time | 68.65 | < 0.0001 | **** | Yes |
| temperature | 1.04 | 0.2473 | ns | No |
| Table S3. D |  |  |  |  |
|  |  |  |  |  |
| Md cwo | | | | |
| Source of Variation | % of total variation | P value | P value summary | Significant? |
| Interaction | 20.91 | < 0.0001 | **** | Yes |
| zeitgeber time | 55.17 | < 0.0001 | **** | Yes |
| temperature | 10.32 | < 0.0001 | **** | Yes |
| Table S3. E |  |  |  |  |

| Md Clk | | | | |
| --- | --- | --- | --- | --- |
| Source of Variation | % of total variation | P value | P value summary | Significant? |
| Interaction | 9.25 | 0.5017 | ns | No |
| zeitgeber time | 57.41 | < 0.0001 | **** | Yes |
| temperature | 2.643 | 0.0399 | * | Yes |
| Table S3. F |  |  |  |  |
|  |  |  |  |  |
| Md cry | | | | |
| Source of Variation | % of total variation | P value | P value summary | Significant? |
| Interaction | 19.04 | 0.2827 | ns | No |
| zeitgeber time | 5.872 | 0.7176 | ns | No |
| temperature | 20.02 | 0.1132 | ns | No |
| Table S3. G |  |  |  |  |
|  |  |  |  |  |
| Md photolyase | | | | |
| Source of Variation | % of total variation | P value | P value summary | Significant? |
| Interaction | 7.272 | 0.9572 | ns | No |
| zeitgeber time | 6.143 | 0.5471 | ns | No |
| temperature | 42.4 | < 0.0001 | **** | Yes |
| Table S3. H |  |  |  |  |
