# Supplementary material for: Daily Activity of the Housefly, Musca domestica, Is Influenced by Temperature Independent of 3′ UTR period Gene Splicing
Source: G3 (Bethesda). 2017 Jun 15;7(8):2637–49. doi: 10.1534/g3.117.042374 (PMC5555469; doi:10.1534/g3.117.042374)
Supplement: Supplementary file 8 [file 2637TableS5.docx]

Tables S5. Statistical comparison of expression analysis in *Drosophila melanogaster* by two-way ANOVA (Graphpad Prims). ****p<0.0001, ***p<0.001, **p<0.01, *p<0.05, ns… non-significant. The tables accompany Figure 6 in the manuscript.

Table S4. A

| Dm per | | | | |
| --- | --- | --- | --- | --- |
| Source of Variation | % of total variation | P value | P value summary | Significant? |
| Interaction | 6.778 | 0.5355 | ns | No |
| zeitgeber time | 54.39 | < 0.0001 | **** | Yes |
| temperature | 15.69 | < 0.0001 | **** | Yes |

Table S4. B

| Dm vri | | | | |
| --- | --- | --- | --- | --- |
| Source of Variation | % of total variation | P value | P value summary | Significant? |
| Interaction | 18.57 | 0.0207 | * | Yes |
| zeitgeber time | 44.95 | < 0.0001 | **** | Yes |
| temperature | 4.195 | 0.0085 | ** | Yes |

Table S4. C

| Dm tim | | | | |
| --- | --- | --- | --- | --- |
| Source of Variation | % of total variation | P value | P value summary | Significant? |
| Interaction | 6.015 | 0.8727 | ns | No |
| zeitgeber time | 58.32 | < 0.0001 | **** | Yes |
| temperature | 6.118 | 0.0006 | *** | Yes |

Table S4. D

| Dm cwo | | | | |
| --- | --- | --- | --- | --- |
| Source of Variation | % of total variation | P value | P value summary | Significant? |
| Interaction | 8.479 | 0.8194 | ns | No |
| zeitgeber time | 23.53 | < 0.0001 | **** | Yes |
| temperature | 29.57 | < 0.0001 | **** | Yes |

Table S4. E

| Dm Clk | | | | |
| --- | --- | --- | --- | --- |
| Source of Variation | % of total variation | P value | P value summary | Significant? |
| Interaction | 19.11 | 0.0005 | *** | Yes |
| zeitgeber time | 49.17 | < 0.0001 | **** | Yes |
| temperature | 8.942 | < 0.0001 | **** | Yes |

Table S4. F

| Dm pdp | | | | |
| --- | --- | --- | --- | --- |
| Source of Variation | % of total variation | P value | P value summary | Significant? |
| Interaction | 26.04 | < 0.0001 | **** | Yes |
| zeitgeber time | 40.9 | < 0.0001 | **** | Yes |
| temperature | 20.14 | < 0.0001 | **** | Yes |

Table S4. G

| Dm cry | | | | |
| --- | --- | --- | --- | --- |
| Source of Variation | % of total variation | P value | P value summary | Significant? |
| Interaction | 9.25 | 0.5017 | ns | No |
| zeitgeber time | 57.41 | < 0.0001 | **** | Yes |
| temperature | 2.643 | 0.0399 | * | Yes |
